# Optimal Estimation of a Signal Generated Using a Dynamical System Modeled with McKean–Vlasov Stochastic Differential Equations

**DOI:** 10.3390/e26060483

**Published:** 2024-05-31

**Authors:** Vasile Dragan, Samir Aberkane

**Affiliations:** 1 Institute of Mathematics “Simion Stoilow” of the Romanian Academy, P.O. Box 1-764, RO-014700 Bucharest, Romania; vasile.dragan@imar.ro; 2 Academy of Romanian Scientists, 3 Ilfov, 050044 Bucharest, Romania; 3 Institute of Mathematics of the Romanian Academy, CRAN, UMR 7039, Campus Sciences, BP 70239, VandÏuvre-les-Nancy CEDEX, 54506 Nancy, France; 4 CNRS, CRAN, UMR 7039, 54500 Nancy, France

**Keywords:** McKean–Vlasov stochastic differential equations, filtering, Riccati differential equations

## Abstract

We consider, in this paper, the problem of state estimation for a class of dynamical systems governed via continuous-time McKean–Vlasov stochastic differential equations. The estimation problem is stated and solved under an $H_{2}$ norm setting. We adopt a Riccati-based approach in order to solve the optimal estimation problem.

## 1. Introduction

McKean–Vlasov stochastic differential equations (SDEs) are a powerful tool used to reflect some kind of mean-field-type interaction phenomena in large-scale dynamical systems. For some pioneering works on theoretical, as well as practical, aspects of such kinds of SDEs, one can refer to [1,2]. As the present article is a control-oriented one, we restricted our bibliographical efforts accordingly. Several contributions have been made over these last few years in different control theoretical fields related to the class of McKean–Vlasov SDEs [3,4,5,6,7,8]. More recently, LQ control problems, as well as LQ games, have gained a lot of interest among the control community; one can refer, for example, to [9,10,11,12,13,14,15]. In these works, the authors studied a class of McKean–Vlasov SDEs that are similar to the one we consider in the present article.

We consider, in this paper, the problem of state estimation for a class of continuous-time, time-varying McKean–Vlasov SDEs. The metric used as an optimality measure of the proposed estimation scheme belongs to the $H_{2}$-type norm setting. More specifically, we introduce a performance criterion expressed in terms of the mean square of the deviation of the estimated signal from the value of the signal that must be estimated. To the best of the authors’ knowledge, it seems that the problem of state estimation for McKean–Vlasov SDEs has not yet been addressed in the literature, or at least it has been addressed only very marginally when compared to the control counterpart. The main objective of the present work is to initiate a filtering research axis for this class of systems. We adopted a Riccati-based approach in order to solve the optimal estimation problem. The present work could be viewed as a generalization of our previous work on $H_{2}$ filtering for Itô-type SDEs [16] in the case where the dynamic equations describing the evolution of the state variable incorporate its mathematical expectation (see Equation (1)).

This paper is organized as follows: Section 2 presents the mathematical model of the considered class of systems and describes the problem setting. Section 3 provides some auxiliary results that will be used in Section 4 in order to obtain the formulae for the computation of the performance value. The main results are presented in Section 5. Some numerical experiments are included in Section 6.

## 2. Problem Formulation

We consider the dynamical system (G) with the state space representation described as follows:

(1) $$\begin{array}{cc} \; & dx\left(t\right)=\left(A_{0}\left(t\right)x\left(t\right)+{\bar{A}}_{0}\left(t\right)E\left[x\left(t\right)\right]\right)dt+\sum_{k=1}^{r}\left(A_{k}\left(t\right)x\left(t\right)+{\bar{A}}_{k}\left(t\right)E\left[x\left(t\right)\right]\right)dw_{k}\left(t\right)+B\left(t\right)dv\left(t\right)\\ \; & dy\left(t\right)=\left(C_{0}\left(t\right)x\left(t\right)+{\bar{C}}_{0}\left(t\right)E\left[x\left(t\right)\right]\right)dt+\sum_{k=1}^{r}\left(C_{k}\left(t\right)x\left(t\right)+{\bar{C}}_{k}\left(t\right)E\left[x\left(t\right)\right]\right)dw_{k}\left(t\right)+D\left(t\right)dv\left(t\right)\\ \; & z\left(t\right)=C_{z}\left(t\right)x\left(t\right)+{\bar{C}}_{z}\left(t\right)E\left[x\left(t\right)\right] \end{array}$$

$t\in R_{+}=\left[0,\infty \right)$, where $x\left(t\right)\in R^{n}$ is the system-state vector, and $y\left(t\right)\in R^{n_{y}}$ are the measurements, while $z\left(t\right)\in R^{n_{z}}$ is the remote signal that must be estimated.

In (1), the sequences ${\left\{w\left(t\right)\right\}}_{t⩾0}$ $\left(w\left(t\right)={\left(w_{1}\left(t\right),\cdots ,w_{r}\left(t\right)\right)}^{⊤}\right)$, ${\left\{v\left(t\right)\right\}}_{t⩾0}$, are stochastic processes defined on a given probability space, (Ω,F,P), satisfying the following assumptions:

**Assumption** **1.**

*(i)*  *${\left\{w\left(t\right)\right\}}_{t⩾0}$ is a r-dimensional, standard Wiener process with a zero mean, and*
*$E\left[\left(w(t)-w(s)\right){\left(w(t)-w(s)\right)}^{⊤}\right]=I_{r}\left(t-s\right),\;\forall \;t⩾s⩾0$, $I_{r}$ is the identity matrix of size r×r;*
*(ii)*  *${\left\{v\left(t\right)\right\}}_{t⩾0}$ is a $m_{v}$-dimensional Wiener process with a zero mean and*
*$E\left[\left(v(t)-v(s)\right){\left(v(t)-v(s)\right)}^{⊤}\right]=V\left(t-s\right),\;\forall \;t⩾s⩾0$, where V⩾0 is a known matrix;*
*(iii)*  *${\left\{w\left(t\right)\right\}}_{t⩾0}$ and ${\left\{v\left(t\right)\right\}}_{t⩾0}$ are independent stochastic processes.*

Throughout the paper, $E\left[\cdot \right]$ stands for the mathematical expectation, and the superscript ⊤ denotes the transposition of a vector or a matrix.

Regarding the coefficients of the system (1), we make the following assumption:

**Assumption** **2.**

*(i)*  *$\left(A_{k}\left(\cdot \right),\;{\bar{A}}_{k}\left(\cdot \right)\right):R_{+}\to R^{n\times n}\times R^{n\times n}$, $\left(C_{k}\left(\cdot \right),\;{\bar{C}}_{k}\left(\cdot \right)\right):R_{+}\to R^{n_{y}\times n}\times R^{n_{y}\times n}$, 0⩽k⩽r, $B\left(\cdot \right):R_{+}\to R^{n\times m_{v}}$, $D\left(\cdot \right):R_{+}\to R^{n_{y}\times m_{v}}$ and $C_{z}:R\to R^{n_{z}\times n}$ are continuous matrix-valued functions that are periodic with period θ>0;*
*(ii)*  *The mean field stochastic linear differential equation (MF-SLDE),*
(2) $$dx\left(t\right)=\left(A_{0}\left(t\right)x\left(t\right)+{\bar{A}}_{0}\left(t\right)E\left[x\left(t\right)\right]\right)dt+\sum_{k=1}^{r}\left(A_{k}\left(t\right)x\left(t\right)+{\bar{A}}_{k}\left(t\right)E\left[x\left(t\right)\right]\right)dw_{k}\left(t\right),\;t\in R_{+}$$
*is exponentially stable in the mean square sense (ESMS).*

Our aim is to design a dynamic linear system ($G_{F}$) named filter that, fed with the measurements y(s) and 0⩽s⩽t generates at its output a signal, $z_{f}\left(t\right)$, which must be *the best estimation* of the remote signal z(t) generated via the dynamical system (G).

In our approach, the family of admissible filters consists of all dynamical systems $G_{F}$ that have the state space representation of the following form:

(3) $$G_{F}:\left\{\begin{array}{c} dx_{F}\left(t\right)=A_{F}\left(t\right)x_{F}\left(t\right)dt+B_{F}\left(t\right)dy\left(t\right)\\ z_{F}\left(t\right)=C_{F}\left(t\right)x_{F}\left(t\right) \end{array}\right.$$

where $x_{F}\left(t\right)\in R^{n_{F}}$ are the state parameters of the filter $G_{F}$. In (3), $A_{F}\left(\cdot \right):R_{+}\to R^{n_{F}\times n_{F}}$, $B_{F}\left(\cdot \right):R_{+}\to R^{n_{F}\times n_{y}}$, $C_{F}\left(\cdot \right):R_{+}\to R^{n_{z}\times n_{F}}$ are arbitrary continuous and θ-periodic matrix-valued functions.

It is worth mentioning that the dimension $n_{F}$ of the state space of an admissible filter (3) is not prefixed.

In what follows, $F_{s}$ denotes the family of all dynamic systems ($G_{F}$) of the form (3) of arbitrary dimension $n_{F}⩾1$ that satisfy the following additional condition:

- The ordinary linear differential equation (OLDE):
  (4) $$\frac{d}{dt}x_{F}\left(t\right)=A_{F}\left(t\right)x_{F}\left(t\right),\;t\in R_{+}$$
  is exponentially stable (ES).

In order to measure the quality of the estimation achieved via a filter ($G_{F}$) $\in F_{s}$, we introduce the following performance criterion:

(5) $$J\left(G_{F}\right)=\lim_{T\to \infty }\frac{1}{T}\int_{t_{0}}^{t_{0}+T}E\left[|z\left(t\right)-z_{F}{\left(t\right)|}^{2}\right]dt$$

In Section 4, we derive an explicit formula for the computation of the value of $J(G_{F})$. We show that this value does not depend either upon the initial time, $t_{0}$, or the initial states of the system (1) and the filter (3). In Section 5, we provide a set of conditions that guarantee the existence of a filter, ${\tilde{G}}_{F}$, minimizing the cost (5) over the set $F_{s}$. We also provide a state-space representation of the optimal filter.

## 3. Some Preliminary Issues

If Assumption 2 (ii) holds, then there exist β⩾1 and α>0, with the property that the solutions x(t) of the MF-SLDE (2) satisfy the following:

(6) $$E\left[{\left|x\left(t\right)\right|}^{2}\right]⩽\beta e^{-\alpha (t-t_{0})}E\left[|x\left(t_{0}\right){|}^{2}\right]$$

for all $t⩾t_{0}⩾0$. From the relation ${\left(E\left[|x(t)|\right]\right)}^{2}⩽E\left[{\left|x\left(t\right)\right|}^{2}\right]$, we deduce via (6) that

(7) $$\lim_{t\to \infty }\left(E\left[|x(t)|\right]\right)=0$$

for any solution, x(t), of (2).

On the other hand, from (2), one obtains confirmation that $t\to E\left[|x(t)|\right]$ solves the OLDE in $R^{n}$:

(8) $$\dot{\xi }\left(t\right)=\left(A_{0}\left(t\right)+{\bar{A}}_{0}\left(t\right)\right)\xi \left(t\right),\;t\in R_{+}$$

Since (8) in an OLDE with periodic coefficients, we deduce via (7) that there exist $\beta_{1}⩾1$ and $\alpha_{1}>0$ such that

(9) $$|E\left[x(t)\right]|⩽\beta_{1}e^{-\alpha_{1}\left(t-t_{0}\right)}\left|E\left[x(t_{0})\right]\right|,\;\forall t⩾t_{0}⩾0$$

If x(t), $t⩾t_{0}⩾0$, is an arbitrary solution to (2), we set the following:

(10a) $$\begin{array}{cc} \; & x^{1}\left(t\right)≜x\left(t\right)-E\left[x\left(t\right)\right] \end{array}$$

(10b) $$\begin{array}{cc} \; & x^{2}\left(t\right)≜E\left[x\left(t\right)\right] \end{array}$$

We have confirmation that $x\left(t\right)=x^{1}\left(t\right)+x^{2}\left(t\right)$ and $E\left[x^{1}\left(t\right){\left(x^{2}\left(t\right)\right)}^{⊤}\right]=0$ for all $t⩾t_{0}⩾0$. Employing the properties of the stochastic Itô-type integrals, we deduce that $t\to {\left({\left(x^{1}\left(t\right)\right)}^{⊤},\;{\left(x^{2}\left(t\right)\right)}^{⊤}\right)}^{⊤}$ solves the following:

(11) $$d\left(\begin{array}{c} x^{1}\left(t\right)\\ x^{2}\left(t\right) \end{array}\right)=A_{0}\left(t\right)\left(\begin{array}{c} x^{1}\left(t\right)\\ x^{2}\left(t\right) \end{array}\right)dt+\sum_{k=1}^{r}A_{k}\left(t\right)\left(\begin{array}{c} x^{1}\left(t\right)\\ x^{2}\left(t\right) \end{array}\right)dw_{k}\left(t\right)$$

where

(12a) $$\begin{array}{cc} \; & A_{0}\left(t\right)=\left(\begin{array}{cc} A_{0}\left(t\right) & 0\\ 0 & {\tilde{A}}_{0}\left(t\right) \end{array}\right) \end{array}$$

(12b) $$\begin{array}{cc} \; & A_{k}\left(t\right)=\left(\begin{array}{cc} A_{k}\left(t\right) & {\tilde{A}}_{k}\left(t\right)\\ 0 & 0 \end{array}\right) \end{array}$$

(12c) $$\begin{array}{cc} \; & {\tilde{A}}_{l}\left(t\right)=A_{l}\left(t\right)+{\bar{A}}_{l}\left(t\right),\;0⩽l⩽r \end{array}$$

1⩽k⩽r. From (6), (9), and (10), we may infer that the SLDE (11) is ESMS if the MF-SLDE (2) is ESMS. In other words, if (2) is ESMS, then there exist $\tilde{\alpha }>0$ and $\tilde{\beta }⩾1$ such that the following applies:

(13) $$E\left[|x^{1}{\left(t\right)|}^{2}\right]+E\left[|x^{2}{\left(t\right)|}^{2}\right]⩽\tilde{\beta }e^{-\tilde{\alpha }\left(t-t_{0}\right)}\left(E\left[|x^{1}\left(t_{0}\right){|}^{2}\right]+E\left[|x^{2}\left(t_{0}\right){|}^{2}\right]\right)$$

for all $t⩾t_{0}⩾0$. Let $X≜S_{n}\times S_{n}$. We recall that, if p⩾2 is a natural number, then $S_{p}\subset R^{p\times p}$ denotes the vector space of symmetric matrices of size p×p. The elements X of the vector space X are pairs of symmetric matrices. In X, we consider the following inner product:

(14) $$\langle X,Y\rangle ≜\mathrm{Tr}\left[X_{1}Y_{1}\right]+\mathrm{Tr}\left[X_{2}Y_{2}\right]$$

for all $X=\left(X_{1},X_{2}\right)$ and $Y=\left(Y_{1},Y_{2}\right)$ from X. Equipped with the inner product (14), X becomes a finite-dimensional, real Hilbert space. Moreover, X is an ordered Hilbert space with the ordering relation ”⩾” induced via the convex cone $X^{+}≜S_{n}^{+}\times S_{n}^{+}$, where $S_{n}^{+}\subset S_{n}$ is the convex cone of positive semidefinite matrices.

We consider the linear operator X→L[X]:X→X defined as follows: $L\left[X\right]=\left(L_{1}\left[X\right],L_{2}\left[X\right]\right)$, where

(15a) $$\begin{array}{cc} \; & L_{1}\left[X\right]=A_{0}\left(t\right)X_{1}+X_{1}A_{0}^{⊤}\left(t\right)+\sum_{k=1}^{r}\left(A_{k}\left(t\right)X_{1}A_{k}^{⊤}\left(t\right)+{\tilde{A}}_{k}\left(t\right)X_{2}{\tilde{A}}_{k}^{⊤}\left(t\right)\right) \end{array}$$

(15b) $$\begin{array}{cc} \; & L_{2}\left[X\right]={\tilde{A}}_{0}\left(t\right)X_{2}+X_{2}{\tilde{A}}_{0}^{⊤}\left(t\right) \end{array}$$

for all $X=\left(X_{1},X_{2}\right)\in X$. Based on the adjoint operator definition, we obtain that the adjoint operator $L^{*}\left[\cdot \right]$ (with respect to the inner product (14)) of the operator L[·] introduced via (15) is given via $L^{*}\left[X\right]=\left(L_{1}^{*}\left[X\right],L_{2}^{*}\left[X\right]\right)$, where:

(16a) $$\begin{array}{cc} \; & L_{1}^{*}\left[X\right]=A_{0}^{⊤}\left(t\right)X_{1}+X_{1}A_{0}\left(t\right)+\sum_{k=1}^{r}A_{k}^{⊤}\left(t\right)X_{1}A_{k}\left(t\right) \end{array}$$

(16b) $$\begin{array}{cc} \; & L_{2}^{*}\left[X\right]={\tilde{A}}_{0}^{⊤}\left(t\right)X_{2}+X_{2}{\tilde{A}}_{0}\left(t\right)+\sum_{k=1}^{r}{\tilde{A}}_{k}^{⊤}\left(t\right)X_{1}{\tilde{A}}_{k}\left(t\right) \end{array}$$

for all $X=\left(X_{1},X_{2}\right)\in X$.

Let $\left(\begin{array}{c} x^{1}\left(t\right)\\ x^{2}\left(t\right) \end{array}\right)$ be an arbitrary solution to the SLDE (11). We set Z(t):= $E\left[\left(\begin{array}{c} x^{1}\left(t\right)\\ x^{2}\left(t\right) \end{array}\right){\left(\begin{array}{c} x^{1}\left(t\right)\\ x^{2}\left(t\right) \end{array}\right)}^{⊤}\right]$. One sees the following:

(17) $$Z\left(t\right)=\left(\begin{array}{cc} Z_{1}\left(t\right) & 0\\ 0 & Z_{2}\left(t\right) \end{array}\right)$$

where

(18) $$Z_{j}\left(t\right)≜E\left[x^{j}\left(t\right){\left(x^{j}\left(t\right)\right)}^{⊤}\right],\;j=1,2$$

On the other hand, the Itô formula applied in the case of the solutions of (11) allows us to deduce that Z(·) solves the OLDE in the space $S_{2n}$:

(19) $$\dot{Z}\left(t\right)=A_{0}\left(t\right)Z\left(t\right)+Z\left(t\right)A_{0}^{⊤}\left(t\right)+\sum_{k=1}^{r}A_{k}\left(t\right)Z\left(t\right)A_{k}^{⊤}\left(t\right)$$

Employing (12), (17), and (18), we obtain confirmation that (19) is equivalent to the following linear differential equation on the Hilbert space X:

(20) $$\dot{Z}\left(t\right)=L\left[Z\left(t\right)\right]$$

where $Z\left(t\right)=\left(Z_{1}\left(t\right),Z_{2}\left(t\right)\right)\in X$.

Based on (13), (18), and (20), together with the equality $x\left(t\right)=x^{1}\left(t\right)+x^{2}\left(t\right)$, we obtain the following:

**Lemma** **1.** *Under Assumptions 1 and 2, the following are equivalent:*

*(i)*  *The MF-SLDE (2) is ESMS;*
*(ii)*  *The SLDE (11) is ESMS;*
*(iii)*  *The linear differential Equation (20) is exponentially stable.*

The next result is used in the development of the next sections.

**Proposition** **1.** *Under Assumptions 1 and 2, the following are equivalent:*

*(i)*  *The MF-SLDE (2) is ESMS;*
*(ii)*  *The SLDE*
(21) $$dx\left(t\right)=A_{0}\left(t\right)x\left(t\right)dt+\sum_{k=1}^{r}A_{k}\left(t\right)x\left(t\right)dw_{k}\left(t\right)$$
*is ESMS, and the OLDE*
(22) $${\dot{x}}^{2}\left(t\right)={\tilde{A}}_{0}\left(t\right)x^{2}\left(t\right)$$
*is exponentially stable.*

**Proof.**  According to the equivalence (i)⇔(iii) from Lemma 1, we may infer that the MF-SLDE (2) is ESMS if and only if the linear differential Equation (20) is exponentially stable. On the other hand, from (15), we deduce via Theorem 2.6.1 from [17] that (20) is a linear differential equation that generates a positive evolution in the ordered space $\left(X,X^{+}\right)$, i.e., if $X\left(t_{0}\right)\in X^{+}$, $\forall t_{0}⩾0$, then $X\left(t\right)\in X^{+}$, $\forall t⩾t_{0}$, where X(·) is a solution to (20).

Invoking the equivalence (i)⇔(vi) from Theorem 2.4.2 of [17], we obtain that (20) is exponentially stable if and only if the non-homogeneous linear differential equation in X,

(23) $$\dot{Y}\left(t\right)+L^{*}\left[Y\left(t\right)\right]+I=0$$

has a unique bounded solution, $t\to \tilde{Y}\left(t\right):R_{+}\to X^{+}$, with the property that there exist positive constants, $\nu_{1},\nu_{2}$, such that

(24) $$0≼\nu_{1}I≼\tilde{Y}\left(t\right)≼\nu_{2}I$$

for all $t\in R_{+}$, where $I=\left(I_{n},I_{n}\right)\in X^{+}$.

Bearing in mind (16), we obtain the following partition of (23) and (24):

(25a) $$\begin{array}{cc} \; & {\dot{\tilde{Y}}}_{1}\left(t\right)+A_{0}^{⊤}\left(t\right){\tilde{Y}}_{1}\left(t\right)+{\tilde{Y}}_{1}\left(t\right)A_{0}\left(t\right)+\sum_{k=1}^{r}A_{k}^{⊤}\left(t\right){\tilde{Y}}_{1}\left(t\right)A_{k}\left(t\right)+I_{n}=0 \end{array}$$

(25b) $$\begin{array}{cc} \; & 0≼\nu_{1}I_{n}≼{\tilde{Y}}_{1}\left(t\right)≼\nu_{2}I_{n} \end{array}$$

$t\in R_{+}$, and

(26a) $$\begin{array}{cc} \; & {\dot{\tilde{Y}}}_{2}\left(t\right)+{\tilde{A}}_{0}^{⊤}\left(t\right){\tilde{Y}}_{2}\left(t\right)+{\tilde{Y}}_{2}\left(t\right){\tilde{A}}_{0}\left(t\right)+\tilde{H}\left(t\right)=0 \end{array}$$

(26b) $$\begin{array}{cc} \; & 0≼\nu_{1}I_{n}≼{\tilde{Y}}_{2}\left(t\right)≼\nu_{2}I_{n} \end{array}$$

$t\in R_{+}$, where $\tilde{H}\left(t\right):=I_{n}+\sum_{k=1}^{r}{\tilde{A}}_{k}^{⊤}\left(t\right){\tilde{Y}}_{1}\left(t\right){\tilde{A}}_{k}\left(t\right)$.

Finally, we take into account that (25) is equivalent to the exponential stability in the mean square sense of the SLDE (21), while (26) is equivalent to the exponential stability of the OLDE (22). Thus, the proof is complete. □

## 4. Computation of the Performance Value

Let x(t) be a solution to (1). Using (10), written for this solution, we obtain the following equivalent form of the state-space representation of the dynamical system (1):

(27a) $$\begin{array}{cc} \; & d\left(\begin{array}{c} x^{1}\left(t\right)\\ x^{2}\left(t\right) \end{array}\right)=A_{0}\left(t\right)\left(\begin{array}{c} x^{1}\left(t\right)\\ x^{2}\left(t\right) \end{array}\right)+\sum_{k=1}^{r}A_{k}\left(t\right)\left(\begin{array}{c} x^{1}\left(t\right)\\ x^{2}\left(t\right) \end{array}\right)dw_{k}\left(t\right)+B\left(t\right)dv\left(t\right) \end{array}$$

(27b) $$\begin{array}{cc} \; & dy\left(t\right)=C_{0}\left(t\right)\left(\begin{array}{c} x^{1}\left(t\right)\\ x^{2}\left(t\right) \end{array}\right)dt+\sum_{k=1}^{r}C_{k}\left(t\right)\left(\begin{array}{c} x^{1}\left(t\right)\\ x^{2}\left(t\right) \end{array}\right)dw_{k}\left(t\right)+D\left(t\right)dv\left(t\right) \end{array}$$

(27c) $$\begin{array}{cc} \; & z\left(t\right)=C_{z}\left(t\right)\left(\begin{array}{c} x^{1}\left(t\right)\\ x^{2}\left(t\right) \end{array}\right) \end{array}$$

$t\in R_{+}$, where $A_{k}\left(t\right)$ and 0⩽k⩽r are described in (12) and

(28a) $$\begin{array}{cc} \; & B\left(t\right)=\left(\begin{array}{c} B(t)\\ 0 \end{array}\right) \end{array}$$

(28b) $$\begin{array}{cc} \; & C_{k}\left(t\right)=\left(\begin{array}{cc} C_{k}\left(t\right) & {\tilde{C}}_{k}\left(t\right) \end{array}\right) \end{array}$$

(28c) $$\begin{array}{cc} \; & {\tilde{C}}_{k}\left(t\right)=C_{k}\left(t\right)+{\bar{C}}_{k}\left(t\right),\;0⩽k⩽r \end{array}$$

(28d) $$\begin{array}{cc} \; & C_{z}\left(t\right)=\left(\begin{array}{cc} C_{z}\left(t\right) & {\tilde{C}}_{z}\left(t\right) \end{array}\right) \end{array}$$

(28e) $$\begin{array}{cc} \; & {\tilde{C}}_{z}\left(t\right)=C_{z}\left(t\right)+{\bar{C}}_{z}\left(t\right) \end{array}$$

Applying a filter, $G_{F}$, from $F_{s}$ to the dynamical system G described by (27), one obtains the following closed-loop system:

(29a) $$\begin{array}{cc} \; & dx_{cl}\left(t\right)={A_{0}}_{cl}\left(t\right)x_{cl}\left(t\right)dt+\sum_{k=1}^{r}{A_{k}}_{cl}\left(t\right)x_{cl}\left(t\right)dw_{k}\left(t\right)+B_{cl}\left(t\right)dv\left(t\right) \end{array}$$

(29b) $$\begin{array}{cc} \; & z_{cl}\left(t\right)=C_{cl}\left(t\right)x_{cl}\left(t\right) \end{array}$$

$t\in R_{+}$, where $x_{cl}\left(t\right)={\left(\begin{array}{ccc} {\left(x^{1}\left(t\right)\right)}^{⊤} & {\left(x^{2}\left(t\right)\right)}^{⊤} & x_{F}^{⊤}\left(t\right) \end{array}\right)}^{⊤}\in R^{2n+n_{F}}$ and:

(30a) $$\begin{array}{cc} \; & {A_{0}}_{cl}\left(t\right)=\left(\begin{array}{cc} A_{0}\left(t\right) & 0\\ B_{F}\left(t\right)C_{0}\left(t\right) & A_{F}\left(t\right) \end{array}\right) \end{array}$$

(30b) $$\begin{array}{cc} \; & {A_{k}}_{cl}\left(t\right)=\left(\begin{array}{cc} A_{k}\left(t\right) & 0\\ B_{F}\left(t\right)C_{k}\left(t\right) & 0 \end{array}\right) \end{array}$$

(30c) $$\begin{array}{cc} \; & B_{cl}\left(t\right)=\left(\begin{array}{c} B(t)\\ B_{F}\left(t\right)D\left(t\right) \end{array}\right) \end{array}$$

(30d) $$\begin{array}{cc} \; & C_{cl}\left(t\right)=\left(\begin{array}{cc} C_{z}\left(t\right) & -C_{F}\left(t\right) \end{array}\right) \end{array}$$

$t\in R_{+}$. Let $t_{0}⩾0$ and ${x_{cl}}_{0}={\left(\begin{array}{ccc} 0^{⊤} & x_{0}^{⊤} & {x_{F}^{⊤}}_{0} \end{array}\right)}^{⊤}\in R^{n}\times R^{n}\times R^{n_{f}}$ be arbitrary but fixed. Let $x_{cl}\left(t\right)=x_{cl}\left(t;t_{0},{x_{cl}}_{0}\right)$ be the solution to (29a) with the initial value $x_{cl}\left(t_{0}\right)={x_{cl}}_{0}$. With these notations, we obtain confirmation that:

$$E\left[|z\left(t\right)-z_{F}{\left(t\right)|}^{2}\right]=E\left[|z_{cl}{\left(t\right)|}^{2}\right]=\mathrm{Tr}\left[C_{cl}\left(t\right)E\left[x_{cl}\left(t\right)x_{cl}^{⊤}\left(t\right)\right]C_{cl}^{⊤}\left(t\right)\right]=\mathrm{Tr}\left[C_{cl}\left(t\right)Y_{cl}\left(t\right)C_{cl}^{⊤}\left(t\right)\right]$$

where

(31) $$Y_{cl}\left(t\right)≜E\left[x_{cl}\left(t\right)x_{cl}^{⊤}\left(t\right)\right]$$

for all $t⩾t_{0}$. Thus, (5) becomes

(32) $$J\left(G_{F}\right)=\lim_{T\to \infty }\frac{1}{T}\int_{t_{0}}^{t_{0}+T}\mathrm{Tr}\left[C_{cl}\left(t\right)Y_{cl}\left(t\right)C_{cl}^{⊤}\left(t\right)\right]dt$$

Using the Itô formula [18] in the case of the stochastic process $x_{cl}\left(t\right)$, one obtains confirmation that $t\to Y_{cl}\left(t\right)$ defined according to (31) is the solution to the following problem with a given initial value in the space $S_{\hat{n}}$ ($\hat{n}:=2n+n_{F}$):

(33a) $$\begin{array}{cc} \; & {\dot{Y}}_{cl}\left(t\right)=L_{cl}\left(t\right)\left[Y_{cl}\left(t\right)\right]+B_{cl}\left(t\right)VB_{cl}^{⊤}\left(t\right),\;t⩾t_{0} \end{array}$$

(33b) $$\begin{array}{cc} \; & Y_{cl}\left(t_{0}\right)={x_{cl}}_{0}{x_{cl}}_{0}^{⊤} \end{array}$$

where $L_{cl}\left(t\right):S_{\hat{n}}\to S_{\hat{n}}$ is described as follows:

(34) $$L_{cl}\left(t\right)\left[Y\right]={A_{0}}_{cl}\left(t\right)Y+Y{A_{0}}_{cl}^{⊤}\left(t\right)+\sum_{k,l=1}^{r}{A_{k}}_{cl}\left(t\right)Y{A_{l}}_{cl}^{⊤}\left(t\right)$$

for all $Y\in S_{\hat{n}}$.

**Remark** **1.** *Even if the closed-loop system (29) works for $t\in R_{+}$, it follows from (30) that, under the assumption* ***H2)*** *ii) and the definition of an admissible filter (3), the coefficients of the closed-loop system are θ-periodic functions. Hence, the coefficients of (29), (33a) and (34) can be extended via periodicity to the whole real axis. Particularly, we have $L_{cl}\left(t+\theta \right)=L_{cl}\left(t\right)$ for all t∈R.*

Regarding the system of SLDE,

(35) $$dx_{cl}\left(t\right)={A_{0}}_{cl}\left(t\right)x_{cl}\left(t\right)dt+\sum_{k=1}^{r}{A_{k}}_{cl}\left(t\right)x_{cl}\left(t\right)dw_{cl}\left(t\right),\;t\in R_{+}$$

obtained from (29a) when $B_{cl}\left(t\right)=0$, $t\in R_{+}$, we have the following:

**Proposition** **2.** *Under the Assumptions 1 and 2, for any filter, $\left(G_{F}\right)$, from $F_{s}$, the corresponding SLDE (35) is ESMS.*

**Proof.**  According to (30a), (30b), we obtain the following partition of (35):

(36a) $$\begin{array}{cc} \; & d\left(\begin{array}{c} x^{1}\left(t\right)\\ x^{2}\left(t\right) \end{array}\right)=A_{0}\left(t\right)\left(\begin{array}{c} x^{1}\left(t\right)\\ x^{2}\left(t\right) \end{array}\right)+\sum_{k=1}^{r}A_{k}\left(t\right)\left(\begin{array}{c} x^{1}\left(t\right)\\ x^{2}\left(t\right) \end{array}\right)dw_{k}\left(t\right) \end{array}$$

(36b) $$\begin{array}{cc} \; & dx_{F}\left(t\right)=\left(A_{F}\left(t\right)x_{F}\left(t\right)+f_{0}\left(t\right)\right)dt+\sum_{k=1}^{r}f_{k}\left(t\right)dw_{k}\left(t\right) \end{array}$$

$t⩾t_{0}$, where we denote the following:

(37) $$f_{k}\left(t\right)≜B_{F}\left(t\right)C_{k}\left(t\right)\left(\begin{array}{c} x^{1}\left(t\right)\\ x^{2}\left(t\right) \end{array}\right),\;0⩽k⩽r$$

Since (36a) is just (11), it follows that its solutions satisfy (13). Thus, $f_{k}\left(t\right)$ defined according to (37) satisfies the assumptions of Theorem 3.6.1 from [17]. Bearing in mind the exponential stability of (4), one concludes via Theorem 3.6.1 from [17] applied to the affine SLDE (36b) that

(38) $$\lim_{t\to \infty }E[|x_{F}\left(t\right){|}^{2}]=0$$

Combining (13) and (38), we may infer that the solutions $x_{cl}\left(t\right)$ of (35) satisfy the following:

$$\lim_{t\to \infty }E[|x_{cl}\left(t\right){|}^{2}]=0$$

Finally, using the fact that (35) has periodic coefficients, we conclude via Theorem 3.2.5 from [17] that the closed-loop SLDE (35) is ESMS. Thus, the proof is complete. □

The operator-valued function $t\to L_{cl}\left(t\right)\left[\cdot \right]$ introduced in (34) defines the following linear differential equation in the Hilbert space $S_{\hat{n}}$:

(39) $$\dot{Y}\left(t\right)=L_{cl}\left(t\right)\left[Y\left(t\right)\right].$$

Let $T(t,t_{0})$ be the linear evolution operator in $S_{\hat{n}}$ generated via the linear differential Equation (39). We recall that, if $Y(t;t_{0},Y_{0})$ is the solution to (39) with the initial value $Y\left(t_{0};t_{0},Y_{0}\right)=Y_{0}$, then $T\left(t,t_{0}\right)\left[Y_{0}\right]=Y\left(t;t_{0},Y_{0}\right)$, for all $t,t_{0}\in R$ and $Y_{0}\in S_{\hat{n}}$.

As a consequence of Proposition 2, we obtain the following:

**Corollary** **1.** *Under the Assumptions 1 and 2, for any filter, $\left(G_{F}\right)$, from $F_{s}$, the corresponding linear differential Equation (39) is exponentially stable, that is, the following applies:*

(40) $$∥T\left(t;t_{0}\right)∥⩽\beta e^{-\alpha (t-t_{0})},\;\forall t⩾t_{0},\;t,t_{0}\in R$$

*where β⩾1 and α>0 do not depend upon t and $t_{0}$.*

**Proof.**  Let $\Phi_{cl}\left(t,t_{0}\right)$ be the fundamental matrix solution to the SLDE (35). It satisfies the following:

$$\begin{array}{cc} \; & d\Phi_{cl}\left(t,t_{0}\right)={A_{0}}_{cl}\left(t\right)\Phi_{cl}\left(t,t_{0}\right)dt+\sum_{k=1}^{r}{A_{k}}_{cl}\left(t\right)\Phi_{cl}\left(t,t_{0}\right)dw_{cl}\left(t\right),\;t⩾t_{0}\\ \; & \Phi_{cl}\left(t_{0},t_{0}\right)=I_{\hat{n}} \end{array}$$

Applying Theorem 3.1.1 from [17] in the special case d=1, we obtain that $T^{*}\left(t,t_{0}\right)\left[I_{\hat{n}}\right]=E\left[\Phi_{cl}^{⊤}\left(t,t_{0}\right)\Phi_{cl}\left(t,t_{0}\right)\right]$, $T^{*}\left(t,t_{0}\right)$ being the adjoint operator of $T(t,t_{0})$ with respect to the inner product of $S_{\hat{n}}$:

(41) $$\langle Y_{1},Y_{2}\rangle =\mathrm{Tr}\left[Y_{1}Y_{2}\right]$$

for any $Y_{1},Y_{2}\in S_{\hat{n}}$. If ${\hat{x}}_{0}\in R^{\hat{n}}$ is an arbitrary vector with $|{\hat{x}}_{0}|=1$, we obtain via Proposition 2 that

$${\hat{x}}_{0}^{⊤}\left(T^{*}\left(t,t_{0}\right)\left[I_{\hat{n}}\right]\right){\hat{x}}_{0}=E\left[|\Phi_{cl}\left(t,t_{0}\right){\hat{x}}_{0}{|}^{2}\right]⩽\beta e^{-\alpha (t-t_{0})}$$

for all $t⩾t_{0}$, $t,t_{0}\in R$, where α>0 and β⩾1 do not depend upon $t,t_{0},{\hat{x}}_{0}$. This leads to $∥T^{*}\left(t,t_{0}\right)\left[I_{\hat{n}}\right]∥⩽\beta e^{-\alpha (t-t_{0})}$, for all $t⩾t_{0}$, $t,t_{0}\in R$, and ∥·∥ being the Euclidian norm of a matrix. Further, we obtain via Corollary 2.1.7 (i) and Theorem 2.1.10 from [17], applied in the case of the linear evolution operator $T^{*}\left(t,t_{0}\right)$, that $∥T^{*}\left(t,t_{0}\right)∥⩽\beta e^{-\alpha (t-t_{0})}$, $\forall t⩾t_{0},t,t_{0}\in R$.

From the equivalence between the norms of the finite-dimensional Hilbert space $S_{\hat{n}}$, we deduce that $∥T^{*}\left(t,t_{0}\right){∥}_{2}⩽\beta e^{-\alpha (t-t_{0})}$, $\forall t⩾t_{0},t,t_{0}\in R$, ${∥\cdot ∥}_{2}$ being the operator norm induced via the norm generated through the inner product (41).

Finally, from the equality $∥T\left(t,t_{0}\right){∥}_{2}={∥T^{*}\left(t,t_{0}\right)∥}_{2}$, we obtain confirmation that (40) holds. This ends the proof. □

**Proposition** **3.** *Under the Assumptions 1 and 2, for each admissible filter, $\left(G_{F}\right)\in F_{s}$, the corresponding non-homogeneous linear differential Equation (33a) has a unique solution $t\to {\tilde{Y}}_{cl}\left(t\right):R\to S_{\hat{n}}$ that is bounded. This solution has the following representation formula:*

(42) $${\tilde{Y}}_{cl}\left(t\right)=\int_{-\infty }^{t}T_{cl}\left(t,s\right)\left[B_{cl}\left(s\right)VB_{cl}^{⊤}\left(s\right)\right]ds$$

*for all t∈R. Additionally, this solution is a θ-periodic function, and it satisfies ${\tilde{Y}}_{cl}\left(t\right)⩾0$ ∀t∈R.*

**Proof.**  First, let us show that the integral from the right-hand side of Equation (42) is convergent. To this end, we use Equation (42) in order to obtain the following:

$$\begin{array}{cc} ∥\int_{-\infty }^{t}T\left(t,s\right)\left[B_{cl}\left(s\right)VB_{cl}^{⊤}\left(s\right)\right]ds∥ & ⩽\int_{-\infty }^{t}∥T\left(t,s\right)∥\cdot ∥B_{cl}\left(s\right)VB_{cl}^{⊤}\left(s\right)∥ds\\ \; & ⩽\beta \gamma \int_{-\infty }^{t}e^{-\alpha (t-s)}ds=\frac{\beta \gamma }{\alpha }<\infty \end{array}$$

∀t∈R, where $\gamma :=\underset{s\in R}{\sup}∥\cdot ∥B_{cl}\left(s\right)VB_{cl}^{⊤}\left(s\right)∥<\infty$. Hence, ${\tilde{Y}}_{cl}\left(t\right)$ is well defined according to (42). The fact that ${\tilde{Y}}_{cl}\left(\cdot \right)$ is a solution to (33a) can be directly deduced using the rule of differentiation of an integral. In order to prove the unicity of the solution defined according to (42), let us assume that ${\hat{Y}}_{cl}\left(\cdot \right):R\to S_{\hat{n}}$ is another bounded solution to (33a). By using the constant-variation formula for any $t>t_{0}$, we obtain the following:

(43) $${\hat{Y}}_{cl}\left(t\right)=T\left(t,t_{0}\right)\left[{\hat{Y}}_{cl}\left(t_{0}\right)\right]+\int_{t_{0}}^{t}T\left(t,s\right)\left[B_{cl}\left(s\right)VB_{cl}^{⊤}\left(s\right)\right]ds$$

Employing (40) again, we obtain the following:

(44) $$∥T\left(t,t_{0}\right)\left[{\hat{Y}}_{cl}\left(t_{0}\right)\right]∥⩽\beta \hat{\gamma }e^{-\alpha (t-t_{0})},\;\forall t>t_{0},\;t,t_{0}\in R$$

where $\hat{\gamma }:=\underset{t_{0}\in R}{\sup}∥{\hat{Y}}_{cl}\left(t_{0}\right)∥$. Letting $t_{0}\to -\infty$ in Equation (43), we obtain via Equation (44) that ${\hat{Y}}_{cl}\left(t\right)=\int_{-\infty }^{t}T\left(t,s\right)\left[B_{cl}\left(s\right)VB_{cl}^{⊤}\left(s\right)\right]={\tilde{Y}}_{cl}\left(t\right)$,∀t∈R. Hence, any bounded solution to (33a) coincides with ${\tilde{Y}}_{cl}\left(\cdot \right)$ defined according to (42).

In order to check the periodicity property of ${\tilde{Y}}_{cl}\left(\cdot \right)$, we write the following:

$$\begin{array}{cc} {\tilde{Y}}_{cl}\left(t+\theta \right) & =\int_{-\infty }^{t+\theta }T\left(t+\theta ,s\right)\left[B_{cl}\left(s\right)VB_{cl}^{⊤}\left(s\right)\right]ds\\ & =\int_{-\infty }^{t}T\left(t+\theta ,s+\theta \right)\left[B_{cl}\left(s+\theta \right)VB_{cl}^{⊤}\left(s+\theta \right)\right]ds\\ \; & =\int_{-\infty }^{t}T\left(t,s\right)\left[B_{cl}\left(s\right)VB_{cl}^{⊤}\left(s\right)\right]ds={\tilde{Y}}_{cl}\left(t\right),\;\forall t\in R \end{array}$$

Thus, the periodicity property of the bounded solution ${\tilde{Y}}_{cl}\left(\cdot \right)$ is confirmed.

Finally, Theorem 2.6.1 from [17], applied in the case of the Lyapunov-type linear differential Equation (33a), allows us to infer that $T\left(t,s\right)\left[B_{cl}\left(s\right)VB_{cl}^{⊤}\left(s\right)\right]\in X^{+}$, ∀t⩾s∈R. This leads to $\int_{-\infty }^{t}T\left(t,s\right)\left[B_{cl}\left(s\right)VB_{cl}^{⊤}\left(s\right)\right]\in X^{+}$, ∀t∈R because X+ is a closed convex cone. Thus, the proof is complete. □

The main result of this section is given in the following theorem:

**Theorem** **1.** *Under the Assumptions 1 and 2, the value of the performance measure (5) achieved using a filter, $\left(G_{F}\right)$, from $F_{s}$ is given as follows:*

(45) $$J\left(G_{F}\right)=\frac{1}{\theta }\int_{0}^{\theta }\mathrm{Tr}\left[C_{cl}\left(s\right){\tilde{Y}}_{cl}\left(s\right)C_{cl}^{⊤}\left(s\right)\right]ds$$

*with ${\tilde{Y}}_{cl}\left(\cdot \right)$ being the unique θ-periodic solution to the non-homogeneous linear differential Equation (33a).*

**Proof.**  Using the version of (5) given in (32), we may write the following:

(46) $$\begin{array}{ccc} J(G_{F}) & = & \lim_{T\to \infty }\frac{1}{T}\int_{t_{0}}^{t_{0}+T}\mathrm{Tr}\left[C_{cl}\left(s\right){\tilde{Y}}_{cl}\left(s\right)C_{cl}^{⊤}\left(s\right)\right]ds+\\ & & +\lim_{T\to \infty }\frac{1}{T}\int_{t_{0}}^{t_{0}+T}\mathrm{Tr}\left[C_{cl}\left(s\right)\left(Y_{cl}\left(s,t_{0}\right)-{\tilde{Y}}_{cl}\left(s\right)\right)C_{cl}^{⊤}\left(s\right)\right]ds. \end{array}$$

Since $t\to Y_{cl}\left(t\right)-{\tilde{Y}}_{cl}\left(t\right)$, $t⩾t_{0}$, is a solution to the linear differential Equation (39), it has the following representation formula:

$$Y_{cl}\left(t\right)-{\tilde{Y}}_{cl}\left(t\right)=T_{cl}\left(t,t_{0}\right)\left[Y_{cl}\left(t_{0},t_{0}\right)-{\tilde{Y}}_{cl}\left(t_{0}\right)\right],\;\forall t⩾t_{0},\;t,t_{0}\in R.$$

Employing (33b) and (40), we obtain the following:

(47) $$∥Y_{cl}\left(t\right)-{\tilde{Y}}_{cl}\left(t\right)∥⩽\beta e^{-\alpha (t-t_{0})}\left(|{x_{cl}}_{0}{|}^{2}+\tilde{c}\right)$$

where $\tilde{c}=\underset{t\in R}{\sup}∥{\tilde{Y}}_{cl}\left(t\right)∥$. Further, (47) allows us to deduce that $\lim_{T\to \infty }\frac{1}{T}\int_{t_{0}}^{t_{0}+T}\mathrm{Tr}\left[C_{cl}\left(s\right)\left(Y_{cl}\left(s,t_{0}\right)-{\tilde{Y}}_{cl}\left(s\right)\right)C_{cl}^{⊤}\left(s\right)\right]ds=0$. Substituting this last equality in (46), we get

(48) $$J\left(G_{F}\right)=\lim_{T\to \infty }\frac{1}{T}\int_{t_{0}}^{t_{0}+T}\mathrm{Tr}\left[C_{cl}\left(s\right){\tilde{Y}}_{cl}\left(s\right)C_{cl}^{⊤}\left(s\right)\right]ds.$$

On the other hand, the boundedness of the function $s\to \mathrm{Tr}\left[C_{cl}\left(s\right){\tilde{Y}}_{cl}\left(s\right)C_{cl}^{⊤}\left(s\right)\right]$ allows us to obtain the equality

(49) $$\lim_{T\to \infty }\frac{1}{T}\int_{t_{0}}^{t_{0}+T}\mathrm{Tr}\left[C_{cl}\left(s\right){\tilde{Y}}_{cl}\left(s\right)C_{cl}^{⊤}\left(s\right)\right]ds=\lim_{T\to \infty }\frac{1}{T}\int_{0}^{T}\mathrm{Tr}\left[C_{cl}\left(s\right){\tilde{Y}}_{cl}\left(s\right)C_{cl}^{⊤}\left(s\right)\right]ds$$

Plugging (49) in (48), we get

$$J\left(G_{F}\right)=\lim_{T\to \infty }\frac{1}{T}\int_{0}^{T}\mathrm{Tr}\left[C_{cl}\left(s\right){\tilde{Y}}_{cl}\left(s\right)C_{cl}^{⊤}\left(s\right)\right]ds=\frac{1}{\theta }\int_{0}^{\theta }\mathrm{Tr}\left[C_{cl}\left(s\right){\tilde{Y}}_{cl}\left(s\right)C_{cl}^{⊤}\left(s\right)\right]ds$$

which is just the right-hand side of (45). For the last equality, we have taken into account that the integration is a θ-periodic function. Thus, the proof is complete. □

**Remark** **2.** *From (45), one sees that the value of the performance $J(G_{F})$ achieved using a filter, $\left(G_{F}\right)$, from $F_{s}$ does not depend on either the initial time instance, $t_{0}$, or the initial states, $x_{0}$ and $x_{F0}$, of the dynamical system (G) and the filter $\left(G_{F}\right)$, respectively.*

## 5. The Optimal Filter

We consider the linear differential equation in $S_{2n}$:

(50) $$\dot{\Pi }\left(t\right)=A_{0}\left(t\right)\Pi \left(t\right)+\Pi \left(t\right)A_{0}^{⊤}\left(t\right)+\sum_{k=1}^{r}A_{k}\left(t\right)\Pi \left(t\right)A_{k}^{⊤}\left(t\right)+B\left(t\right)VB^{⊤}\left(t\right),\;t\in R$$

or, equivalently,

(51) $$\dot{\Pi }\left(t\right)=L\left(t\right)\left[\Pi \left(t\right)\right]+B\left(t\right)VB^{⊤}\left(t\right)$$

where $L\left(t\right)\left[\cdot \right]:S_{2n}\to S_{2n}$ is defined in (15).

**Proposition** **4.** *Under the Assumptions 1 and 2, the linear differential Equation (50) admits a unique bounded solution, $t\to \tilde{\Pi }\left(t\right):R\to S_{2n}$. Additionally, it is a θ-periodic function, and it has the following structure:*

(52) $$\tilde{\Pi }\left(t\right)=\left(\begin{array}{cc} \Pi_{c}\left(t\right) & 0\\ 0 & 0 \end{array}\right),\;t\in R$$

*where $\Pi_{c}\left(\cdot \right)$ is the unique bound of the R solution to the linear differential equation in $S_{n}$:*

(53) $${\dot{\Pi }}_{c}\left(t\right)=A_{0}\left(t\right)\Pi_{c}\left(t\right)+\Pi_{c}\left(t\right)A_{0}^{⊤}\left(t\right)+\sum_{k=1}^{r}A_{k}\left(t\right)\Pi_{c}\left(t\right)A_{k}^{⊤}\left(t\right)+B\left(t\right)VB^{⊤}\left(t\right),\;t\in R$$

**Proof.**  We look for a solution, $\tilde{\Pi }\left(\cdot \right)$, to the Equation (50), which is bounded on R and has the following structure:

$$\tilde{\Pi }\left(t\right)=\left(\begin{array}{cc} \Pi_{11}\left(t\right) & \Pi_{12}\left(t\right)\\ \Pi_{12}^{⊤}\left(t\right) & \Pi_{22}\left(t\right) \end{array}\right),\;t\in R$$

with $\Pi_{ij}\left(t\right)\in S_{n}$. Employing (12), together with (28a), we obtain the following partition of the Equation (50) written for the solution $\tilde{\Pi }\left(\cdot \right)$:

(54a) $$\begin{array}{cc} \; & {\dot{\Pi }}_{11}\left(t\right)=A_{0}\left(t\right)\Pi_{11}\left(t\right)+\Pi_{11}\left(t\right)A_{0}^{⊤}\left(t\right)+\sum_{k=1}^{r}\left(A_{k}\left(t\right)\Pi_{11}\left(t\right)A_{k}^{⊤}\left(t\right)+{\tilde{A}}_{k}\left(t\right)\Pi_{22}\left(t\right){\tilde{A}}_{k}^{⊤}\left(t\right)\right) \end{array}$$

(54b) $$\begin{array}{c} +B\left(t\right)VB^{⊤}\left(t\right) \end{array}$$

(54c) $$\begin{array}{cc} \; & {\dot{\Pi }}_{12}\left(t\right)=A_{0}\left(t\right)\Pi_{12}\left(t\right)+\Pi_{12}\left(t\right){\tilde{A}}_{0}^{⊤}\left(t\right) \end{array}$$

(54d) $$\begin{array}{cc} \; & {\dot{\Pi }}_{22}\left(t\right)={\tilde{A}}_{0}\left(t\right)\Pi_{22}\left(t\right)+\Pi_{22}\left(t\right){\tilde{A}}_{0}^{⊤}\left(t\right) \end{array}$$

t∈R. If the MF-SLDE (2) is ESMS, then according to (i)→(ii) from Proposition 1, we obtain confirmation that the OLDE (22) is ES. In this case, the linear differential Equation (54d) has a unique bound in the R solution, and this solution is $\Pi_{22}\left(t\right)=0$, t∈R.

Hence, (54a) reduces to

(55) $${\dot{\Pi }}_{11}\left(t\right)=A_{0}\left(t\right)\Pi_{11}\left(t\right)+\Pi_{11}\left(t\right)A_{0}^{⊤}\left(t\right)+\sum_{k=1}^{r}A_{k}\left(t\right)\Pi_{11}\left(t\right)A_{k}^{⊤}\left(t\right)+B\left(t\right)VB^{⊤}\left(t\right)$$

Invoking again the implication (i)→(ii) from Proposition 1, we deduce that the SLDE (21) is ESMS. In this case, the linear differential equation of the Lyapunov type associated with (21),

(56) $$\dot{X}\left(t\right)=A_{0}\left(t\right)X\left(t\right)+X\left(t\right)A_{0}^{⊤}\left(t\right)+\sum_{k=1}^{r}A_{k}\left(t\right)X\left(t\right)A_{k}^{⊤}\left(t\right)$$

defines a positive evolution, and it is exponentially stable in $S_{n}$. Hence, the non-homogeneous Equation (55) has a unique bound in the R solution, and this solution is a θ-periodic function that satisfies $\Pi_{11}\left(t\right)⩾0$, ∀t∈R.

Since (55) coincides with (53), it follows that $\Pi_{11}\left(t\right)=\Pi_{c}\left(t\right)$ and ∀t∈R, which is the unique bound in the R solution to Equation (53). The need to search for the bound in the R solutions of (54c) remains. First, let us remark that, if the SLDE (21) is ESMS, then the OLDE

(57) $$\dot{x}\left(t\right)=A_{0}\left(t\right)x\left(t\right)$$

is exponentially stable. Let $\Psi_{A_{0}}\left(\theta \right)$ be the monodromy matrix of the differential Equation (57). That is, $\Psi_{A_{0}}\left(\theta \right)=\Phi_{A_{0}}\left(\theta ,0\right)$, where $\Phi_{A_{0}}\left(\cdot ,0\right)$ is the matrix solution to (57), which satisfies $\Phi_{A_{0}}\left(0,0\right)=I_{n}$. The eigenvalues of the monodromy matrix $\Psi_{A_{0}}\left(\theta \right)$ are located in the disk |λ|<1 because (57) is ES. Similarly, the eigenvalues of the monodromy matrix $\Psi_{{\tilde{A}}_{0}}\left(\theta \right)$ of the differential Equation (22) are located in the disk |λ|<1. If Ψ(θ) is the monodromy operator of the Equation (54c), it is obtained via $\Psi \left(\theta \right)Y=\Psi_{A_{0}}\left(\theta \right)Y\Psi_{{\tilde{A}}_{0}}^{⊤}\left(\theta \right)$.

The eigenvalues of Ψ(θ) are, then, of the form $\lambda =\mu \bar{\nu }$, where μ is an eigenvalue of $\Psi_{A_{0}}\left(\theta \right)$, and ν is an eigenvalue of $\Psi_{{\tilde{A}}_{0}}\left(\theta \right)$. Hence, $|\lambda |=|\mu \bar{\nu }|<1$. We conclude that Equation (54c) may have only one bound in the R solution (see, for example, Theorem 2.3.7 from [17]). This means that $\Pi_{12}\left(t\right)=0$, t∈R is the unique bound in the R solution to (54c). This ends the proof. □

Let

(58) $$\tilde{R}\left(t\right)≜D\left(t\right)VD^{⊤}\left(t\right)+\sum_{k=1}^{r}C_{k}\left(t\right)\tilde{\Pi }\left(t\right)C_{k}^{⊤}\left(t\right),\;t\in R$$

Using (28b) and (30), we obtain confirmation that (58) becomes

(59) $$\tilde{R}\left(t\right)≜D\left(t\right)VD^{⊤}\left(t\right)+\sum_{k=1}^{r}C_{k}\left(t\right)\Pi_{c}\left(t\right)C_{k}^{⊤}\left(t\right),\;t\in R$$

Now, we introduce the following forward Riccati differential equation (F-RDE):

(60) $$\dot{Y}\left(t\right)=A_{0}\left(t\right)Y\left(t\right)+Y\left(t\right)A_{0}^{⊤}\left(t\right)-\left(Y\left(t\right)C_{0}^{⊤}\left(t\right)+\tilde{L}\left(t\right)\right){\tilde{R}}^{-1}\left(t\right)\left(C_{0}\left(t\right)Y\left(t\right)+{\tilde{L}}^{⊤}\left(t\right)\right)+\tilde{M}\left(t\right),\;t\in R$$

where

(61a) $$\begin{array}{cc} \; & \tilde{L}\left(t\right)=B\left(t\right)VD^{⊤}\left(t\right)+\sum_{k=1}^{r}A_{k}\left(t\right)\tilde{\Pi }\left(t\right)C_{k}^{⊤}\left(t\right) \end{array}$$

(61b) $$\begin{array}{cc} \; & \tilde{M}\left(t\right)=B\left(t\right)VB^{⊤}\left(t\right)+\sum_{k=1}^{r}A_{k}\left(t\right)\tilde{\Pi }\left(t\right)A_{k}^{⊤}\left(t\right) \end{array}$$

Employing (12), (28a)–(28c), and (52), we obtain confirmation that:

$$\tilde{L}\left(t\right)=\left(\begin{array}{c} {\tilde{L}}_{1}\left(t\right)\\ 0 \end{array}\right),\;\tilde{M}\left(t\right)=\left(\begin{array}{cc} {\tilde{M}}_{1}\left(t\right) & 0\\ 0 & 0 \end{array}\right)$$

with

(62a) $$\begin{array}{cc} \; & {\tilde{L}}_{1}\left(t\right)=B\left(t\right)VD^{⊤}\left(t\right)+\sum_{k=1}^{r}A_{k}\left(t\right)\Pi_{c}\left(t\right)C_{k}^{⊤}\left(t\right) \end{array}$$

(62b) $$\begin{array}{cc} \; & {\tilde{M}}_{1}\left(t\right)=B\left(t\right)VB^{⊤}\left(t\right)+\sum_{k=1}^{r}A_{k}\left(t\right)\Pi_{c}\left(t\right)A_{k}^{⊤}\left(t\right) \end{array}$$

We recall now the following definition:

**Definition** **1.** *A globally defined solution, $Y\left(\cdot \right):R\to S_{2n}$, of the F-RDE (60) is named a stabilizing solution if the OLDE on $R^{2n}$,*

(63) $$\dot{x}\left(t\right)=\left(A_{0}\left(t\right)+K_{s}\left(t\right)C_{0}\left(t\right)\right)x\left(t\right)$$

*is exponentially stable, where*

(64) $$K_{s}\left(t\right):=-\left(Y_{s}\left(t\right)C_{0}^{⊤}\left(t\right)+\tilde{L}\left(t\right)\right){\tilde{R}}^{-1}\left(t\right),\;t\in R$$

**Remark** **3.**

*(a)*  *From (58) and (61), one obtains confirmation that*
(65) $$\left(\begin{array}{cc} \tilde{M}\left(t\right) & \tilde{L}\left(t\right)\\ {\tilde{L}}^{⊤}\left(t\right) & \tilde{R}\left(t\right) \end{array}\right)⩾0,\;\forall t\in R$$
*(b)*  *Via a direct calculation, one obtains confirmation that the F-RDE (60), verified via its stabilizing solution, $Y_{s}\left(\cdot \right)$, can be written in the following form:*
(66) $${\dot{Y}}_{s}\left(t\right)=\left(A_{0}\left(t\right)+K_{s}\left(t\right)C_{0}\left(t\right)\right)Y_{s}\left(t\right)+Y_{s}\left(t\right){\left(A_{0}\left(t\right)+K_{s}\left(t\right)C_{0}\left(t\right)\right)}^{⊤}+Q\left(t\right),\;t\in R$$
  *where $Q\left(t\right)=\left(\begin{array}{cc} I_{2n} & K_{s}\left(t\right) \end{array}\right)\left(\begin{array}{cc} \tilde{M}\left(t\right) & \tilde{L}\left(t\right)\\ {\tilde{L}}^{⊤}\left(t\right) & \tilde{R}\left(t\right) \end{array}\right)\left(\begin{array}{c} I_{2n}\\ K_{s}^{⊤}\left(t\right) \end{array}\right)$, t∈R. In this case, (66) may have only one θ-periodic solution, and such a solution is a positive semidefinite because the OLDE (63) is exponentially stable, and (65) holds. Hence, the stabilizing and bounded solution to (60), if any, satisfies the following condition:*
(67) $$Y_{s}\left(t\right)⩾0,\;t\in R$$
*(c)*  *If the SLDE (21) is ESMS, then the OLDE on $R^{n}$, $\dot{x}\left(t\right)=A_{0}\left(t\right)x\left(t\right)$, is ES. Thus, if the Assumptions 1 and 2 hold, we may infer, via the implication (i)→(ii) from Proposition 1 and Formula (12a), that the OLDE on $R^{2n}$,*
(68) $$\dot{x}\left(t\right)=A_{0}\left(t\right)x\left(t\right),\;t\in R$$
  *is ES.*
*(d)*  *Substracting (60) (written for Y(t) and replaced with $Y_{s}\left(t\right)$) from (50) (written for Π(t) and replaced with $\tilde{\Pi }\left(t\right)$), we obtain confirmation that $\Delta \left(\cdot \right):=\tilde{\Pi }\left(\cdot \right)-Y_{s}\left(\cdot \right)$ is a bound in the R solution to the following linear differential equation:*
(69) $${\dot{\Delta }}_{s}\left(t\right)=A_{0}\left(t\right)\Delta \left(t\right)+\Delta \left(t\right)A_{0}^{⊤}\left(t\right)+K_{s}\left(t\right)\tilde{R}\left(t\right)K_{s}^{⊤}\left(t\right)$$
*Bearing in mind that the OLDE (68) is ES and $\tilde{R}\left(t\right)>0$ for all t∈R, we conclude that (69) has a unique bound in the R solution, and additionally, this solution is positive and semidefinite. Under the Assumptions 1 and 2, the stabilizing and bounded solution to the F-RDE, if any, necessarily satisfies the following constraint:*
(70) $$0⩽Y_{s}\left(t\right)⩽\tilde{\Pi }\left(t\right)$$
  *for all t∈R.*
*(e)*  *Let $\left(\begin{array}{cc} Y_{s}^{1}\left(t\right) & Y_{s}^{2}\left(t\right)\\ {\left(Y_{s}^{2}\right)}^{⊤}\left(t\right) & Y_{s}^{3}\left(t\right) \end{array}\right)$ be the partition of the matrix $Y_{s}\left(t\right)$, such that $Y_{s}^{1}\left(t\right)\in S_{n}$, $Y_{s}^{3}\left(t\right)\in S_{n}$. Employing (52) and (70), we conclude that we necessarily have $Y_{s}^{3}\left(t\right)=0$ and $Y_{s}^{2}\left(t\right)=0$, t∈R. Thus, under the Assumptions 1 and 2, the bounded and stabilizing solution $Y_{s}\left(\cdot \right)$ of the F-RDE (60), if any, has the following structure:*
(71) $$Y_{s}\left(t\right)=\left(\begin{array}{cc} Y_{s}^{1}\left(t\right) & 0\\ 0 & 0 \end{array}\right)$$
  *where $Y_{s}^{1}\left(\cdot \right)$ solves the F-RDE of the lower dimension,*
(72) $${\dot{Y}}^{1}\left(t\right)=A_{0}\left(t\right)Y^{1}\left(t\right)+Y^{1}\left(t\right)A_{0}^{⊤}\left(t\right)-\left(Y^{1}\left(t\right)C_{0}^{⊤}\left(t\right)+{\tilde{L}}_{1}\left(t\right)\right){\tilde{R}}^{-1}\left(t\right)★+{\tilde{M}}_{1}\left(t\right),\;t\in R$$
  *where ${\tilde{M}}_{1}\left(t\right)$ and ${\tilde{L}}_{1}\left(t\right)$ are defined in (62).*

By adapting Definition 1 to the case of the F-RDE (72), we say that a solution, $Y_{s}^{1}\left(\cdot \right)$, of (72) is a stabilizing solution if the following OLDE on $R^{n}$,

(73) $$\dot{x}\left(t\right)=\left(A_{0}\left(t\right)+K_{s}^{1}\left(t\right)C_{0}\left(t\right)\right)x\left(t\right)$$

is ES, where

(74) $$K_{s}^{1}\left(t\right):=-\left(Y_{s}^{1}\left(t\right)C_{0}^{⊤}\left(t\right)+{\tilde{L}}_{1}\left(t\right)\right){\tilde{R}}^{-1}\left(t\right),\;t\in R$$

The following result establishes a relationship between the θ-periodic and stabilizing solution to the F-RDE (72) and the θ-periodic and stabilizing solution to the F-RDE (60).

**Lemma** **2.** *Assume that the Assumptions 1 and 2 are fulfilled. If $Y_{s}^{1}\left(\cdot \right)$ is the unique θ-periodic and stabilizing solution to the F-RDE (72), then $Y_{s}\left(\cdot \right)$, constructed as in (71), is the unique θ-periodic and stabilizing solution to the F-RDE (60). In this case, (64) becomes*

(75) $$K_{s}\left(t\right)=\left(\begin{array}{c} K_{s}^{1}\left(t\right)\\ 0 \end{array}\right)$$

*where $K_{s}^{1}\left(t\right)$ is introduced via (74).*

**Proof.**  Bearing in mind (12a), (28b), (28c), and (75), we obtain confirmation that $A_{0}\left(t\right)+K_{s}\left(t\right)C_{0}\left(t\right)=\left(\begin{array}{cc} A_{0}\left(t\right)+K_{s}^{1}\left(t\right)C_{0}\left(t\right) & K_{s}^{1}\left(t\right){\tilde{C}}_{0}\left(t\right)\\ 0 & {\tilde{A}}_{0}\left(t\right) \end{array}\right)$. This shows that (63) is exponentially stable because both (22) and (73) are exponentially stable. Hence, the proof is complete. □

**Remark** **4.** *Under the Assumptions 1 and 2, in order to test the existence of the stabilizing solution to the F-RDE (60), it is sufficient to test the existence of the stabilizing solution to the F-RDE of lower dimension (72). To this end, one can use the set of necessary and sufficient existence conditions proposed in [16].*

The main result of this work is given below:

**Theorem** **2.** *Assume the following:*

*(a)*  *The Assumptions 1 and 2 are fulfilled.*
*(b)*  *$\tilde{R}\left(t\right)>0,\;\forall t\in R$ and the F-RDE (72) have a stabilizing solution, $Y_{s}^{1}\left(\cdot \right)$, which is θ-periodic.*

*Let $K_{s}^{1}\left(\cdot \right)$ be the feedback gain associated with the stabilizing solution $Y_{s}^{1}\left(\cdot \right)$ via (74). Let $Y_{s}\left(t\right)\in S_{2n}$ and $K_{s}\left(t\right)\in R^{2n\times n_{y}}$, t∈R, be defined as in (71) and (75), respectively. Consider the filter ${\tilde{G}}_{F}$ with dimension $n_{F}=2n$ and the following state space representation:*

(76a) $$\begin{array}{cc} \; & dx_{F}\left(t\right)=\left(A_{0}\left(t\right)+K_{s}\left(t\right)C_{0}\left(t\right)\right)x_{F}\left(t\right)dt-K_{s}\left(t\right)dy\left(t\right) \end{array}$$

(76b) $$\begin{array}{cc} \; & z_{F}\left(t\right)=C_{z}\left(t\right)x_{F}\left(t\right) \end{array}$$

*$t\in R_{+}$. Under the considered assumptions, the filter ${\tilde{G}}_{F}$ lies in $F_{s}$ and minimizes the cost functional (5) over $F_{s}$. The minimal value achieved via the performance index (5) is as follows:*

(77) $$J\left({\tilde{G}}_{F}\right)=\frac{1}{\theta }\int_{0}^{\theta }\mathrm{Tr}\left[C_{z}\left(t\right)Y_{s}^{1}\left(t\right)C_{z}^{⊤}\left(t\right)\right]dt$$

**Proof.**  The fact that the filter (76) lies in $F_{s}$ is obvious because, in this case, the corresponding system (4) coincides with (63), and therefore, it is ES. In order to check that the filter (76) provides the minimal value of the cost (5), let us consider an arbitrary but fixed filter, $G_{F}\in F_{s}$. Let $\left(\begin{array}{cc} Y_{11}\left(t\right) & Y_{12}\left(t\right)\\ Y_{12}^{⊤}\left(t\right) & Y_{22}\left(t\right) \end{array}\right)$, t∈R, be the partition of the θ-periodic solution ${\tilde{Y}}_{cl}$ of the corresponding linear differential equation of type (33a), such that $Y_{11}\left(t\right)\in S_{2n}$ and $Y_{22}\left(t\right)\in S_{n_{F}}$. Based on (30a)–(30c), we obtain the following partition of the Equation (33a) written for its θ-periodic solution:

(78a) $$\begin{array}{cc} \; & {\dot{Y}}_{11}\left(t\right)=A_{0}\left(t\right)Y_{11}\left(t\right)+Y_{11}\left(t\right)A_{0}^{⊤}\left(t\right)+\sum_{k=1}^{r}A_{k}\left(t\right)Y_{11}\left(t\right)A_{k}^{⊤}\left(t\right)+B\left(t\right)VB^{⊤}\left(t\right)\\ \; & {\dot{Y}}_{12}\left(t\right)=A_{0}\left(t\right)Y_{12}\left(t\right)+Y_{11}\left(t\right)C_{0}^{⊤}\left(t\right)B_{F}^{⊤}\left(t\right)+Y_{12}\left(t\right)A_{F}^{⊤}\left(t\right)+\sum_{k=1}^{r}A_{k}\left(t\right)Y_{11}\left(t\right)C_{k}^{⊤}\left(t\right)B_{F}^{⊤}\left(t\right) \end{array}$$

(78b) $$\begin{array}{cc} \; & +B\left(t\right)VD^{⊤}\left(t\right)B_{F}^{⊤}\left(t\right)\\ \; & {\dot{Y}}_{22}\left(t\right)=B_{F}\left(t\right)C_{0}\left(t\right)Y_{12}\left(t\right)+A_{F}\left(t\right)Y_{22}\left(t\right)+Y_{12}^{⊤}\left(t\right)C_{0}^{⊤}\left(t\right)B_{F}^{⊤}\left(t\right)+Y_{22}\left(t\right)A_{F}^{⊤}\left(t\right) \end{array}$$

(78c) $$\begin{array}{cc} \; & +\sum_{k=1}^{r}B_{F}\left(t\right)C_{k}\left(t\right)Y_{11}\left(t\right)C_{k}^{⊤}\left(t\right)B_{F}^{⊤}\left(t\right)+B_{F}\left(t\right)D\left(t\right)VD^{⊤}\left(t\right)B_{F}^{⊤}\left(t\right). \end{array}$$

One sees that the Equation (78a) coincides with (50). From the uniqueness of the θ-periodic solution to the non-homogeneous, Lyapunov-type linear differential equation associated with a mean square, exponentially stable, stochastic linear differential equation, we deduce that $Y_{11}\left(t\right)=\tilde{\Pi }\left(t\right),\;\forall t\in R$.

Let $U\left(t\right)≜\left(\begin{array}{cc} \tilde{\Pi }\left(t\right)-Y_{s}\left(t\right) & Y_{12}\left(t\right)\\ Y_{12}^{⊤}\left(t\right) & Y_{22}\left(t\right) \end{array}\right),\;t\in R$. Via a direct calculation involving (60), together with (78) (for which $Y_{11}\left(\cdot \right)$ is replaced with $\tilde{\Pi }\left(\cdot \right)$), we obtain confirmation that U(·) is a θ-periodic solution to the following Lyapunov-type differential equation:

(79) $$\dot{U}\left(t\right)={A_{0}}_{cl}\left(t\right)U\left(t\right)+U\left(t\right){A_{0}}_{cl}^{⊤}\left(t\right)+{\overset{ˇ}{B}}_{cl}\left(t\right)\tilde{R}\left(t\right){\overset{ˇ}{B}}_{cl}^{⊤}\left(t\right)$$

where ${\overset{ˇ}{B}}_{cl}\left(t\right)≜\left(\begin{array}{c} K_{s}\left(t\right)\\ -B_{F}\left(t\right) \end{array}\right)$, and $\tilde{R}\left(t\right)$ was introduced in (58). Since the differential Equation (39) defines a positive evolution on the ordered Hilbert space $\left(S_{\hat{n}},S_{\hat{n}}^{+}\right)$, and (40) holds, we deduce via the equivalence (i)⇔(vi) in Theorem 2.4.2 from [17], applied in the case of the linear differential Equation (39), that there exists a $C^{1}$-matrix-valued function $t\to S\left(t\right):R\to S_{\hat{n}}$ satisfying

$$\dot{S}\left(t\right)+{A_{0}}_{cl}^{⊤}\left(t\right)S\left(t\right)+S\left(t\right){A_{0}}_{cl}\left(t\right)⩽-I_{\hat{n}}$$

and

$$0<\nu_{1}I_{\hat{n}}⩽S\left(t\right)⩽\bar{\nu }<\nu_{2}I_{\hat{n}},\;\forall t\in R.$$

This means that the OLDE on $R^{\hat{n}}$,

$${\dot{x}}_{cl}\left(t\right)={A_{0}}_{cl}\left(t\right)x_{cl}\left(t\right),\;t\in R$$

is ES. This allows us to conclude that (79) has a unique θ-periodic and positive semidefinite solution. This leads to the following:

(80) U(t)⩾0,t∈R.

Further, we rewrite (45) as follows:

(81) $$J\left(G_{F}\right)=\frac{1}{\theta }\int_{0}^{\theta }\mathrm{Tr}\left[C_{z}\left(t\right)Y_{s}\left(t\right)C_{z}^{⊤}\left(t\right)\right]dt+\frac{1}{\theta }\int_{0}^{\theta }\mathrm{Tr}\left[C_{cl}\left(t\right)U\left(t\right)C_{cl}^{⊤}\left(t\right)\right]dt$$

Combining (80) and (81), we deduce that

(82) $$J\left(G_{F}\right)⩾\frac{1}{\theta }\int_{0}^{\theta }\mathrm{Tr}\left[C_{z}\left(t\right)Y_{s}\left(t\right)C_{z}^{⊤}\left(t\right)\right]dt$$

for all $G_{F}\in F_{s}$. It remains to show that, in the special case of the filter ${\tilde{G}}_{F}$ introduced via (76), the inequality (82) becomes equal. To this end, let us remark that, in the case of the filter (76), we have the following:

(83) $$C_{cl}\left(t\right)U\left(t\right)C_{cl}^{⊤}\left(t\right)=C_{z}\left(t\right)\left(\begin{array}{cc} I_{2n} & -I_{2n} \end{array}\right)U\left(t\right){\left(\begin{array}{cc} I_{2n} & -I_{2n} \end{array}\right)}^{⊤}C_{z}^{⊤}\left(t\right)=C_{z}\left(t\right){\hat{U}}_{11}\left(t\right)C_{z}^{⊤}\left(t\right)$$

with ${\hat{U}}_{11}\left(t\right)$ being the 11-block of the matrix $\hat{U}\left(t\right)=WU\left(t\right)W^{⊤}$, $W=\left(\begin{array}{cc} I_{2n} & -I_{2n}\\ 0 & I_{2n} \end{array}\right)$. Via a direct calculation involving the differential Equation (79), we obtain confirmation that $t\to {\hat{U}}_{11}\left(t\right)$ is the θ-periodic solution to the linear differential equation

(84) $$\frac{d}{dt}{\hat{U}}_{11}\left(t\right)=\left(A_{0}\left(t\right)+K_{s}\left(t\right)C_{0}\left(t\right)\right){\hat{U}}_{11}\left(t\right)+{\hat{U}}_{11}\left(t\right){\left(A_{0}\left(t\right)+K_{s}\left(t\right)C_{0}\left(t\right)\right)}^{⊤}.$$

Since the differential Equation (63) is ES, we conclude that the differential Equation (84) has a unique θ-periodic solution, namely ${\hat{U}}_{11}\left(t\right)=0$, t∈R.

Plugging this equality into (83), we deduce that, in the case of the filter (76), the equality (81) is reduced to (77). Thus, the proof is complete. □

## 6. Numerical Experiments

It appears from Theorem 2 that the synthesis of the optimal $H_{2}$-filter for system (1) relies on the resolution to the periodic F-RDE (72) whose coefficients depend on the unique θ-periodic solution to the non-homogeneous, Lyapunov-type linear differential Equation (50). Hence, in order to synthesize the optimal filter of type (76), one has first to solve the equations (50) and (72), respectively.

In what follows, we generate an artificial θ-periodic linear system of the McKean–Vlasov type. To this end, we generalize the procedure proposed in [19]. First, we generate a stochastic linear time-invariant (LTI) system of the McKean–Vlasov type:

(85) $$\left\{\begin{array}{c} dx\left(t\right)=\left(A_{0}x\left(t\right)+{\bar{A}}_{0}E\left[x\left(t\right)\right]\right)dt+\left(A_{1}x\left(t\right)+{\bar{A}}_{1}E\left[x\left(t\right)\right]\right)dw_{k}\left(t\right)+Bdv\left(t\right)\\ dy\left(t\right)=\left(C_{0}x\left(t\right)+{\bar{C}}_{0}E\left[x\left(t\right)\right]\right)dt+\left(C_{1}x\left(t\right)+{\bar{C}}_{1}E\left[x\left(t\right)\right]\right)dw_{k}\left(t\right)+Ddv\left(t\right)\\ z\left(t\right)=C_{z}x\left(t\right)+{\bar{C}}_{z}E\left[x\left(t\right)\right] \end{array}\right.$$

Next, the LTI system (85) is transformed into a stochastic-periodic linear system via the change in the system coordinate $\bar{x}\left(t\right)=G\left(t\right)x\left(t\right)$, where

$$G\left(t\right)=\mathrm{diag}\left(\begin{array}{ccc} \hat{G}\left(t\right) & \cdots & \hat{G}\left(t\right) \end{array}\right),\;\hat{G}\left(t\right)=\left[\begin{array}{cc} \cos(wt) & \sin(wt)\\ -\sin(wt) & \cos(wt) \end{array}\right]$$

for a given w>0. This results in the following stochastic θ-periodic McKean–Vlasov-type system:

(86) $$\left\{\begin{array}{c} d\bar{x}\left(t\right)=\left(A_{0}\left(t\right)\bar{x}\left(t\right)+{\bar{A}}_{0}\left(t\right)E\left[\bar{x}\left(t\right)\right]\right)dt+\left(A_{1}\left(t\right)\bar{x}\left(t\right)+{\bar{A}}_{1}\left(t\right)E\left[\bar{x}\left(t\right)\right]\right)dw_{k}\left(t\right)+B\left(t\right)dv\left(t\right)\\ dy\left(t\right)=\left(C_{0}\left(t\right)\bar{x}\left(t\right)+{\bar{C}}_{0}\left(t\right)E\left[\bar{x}\left(t\right)\right]\right)dt+\left(C_{1}\left(t\right)\bar{x}\left(t\right)+{\bar{C}}_{1}\left(t\right)E\left[\bar{x}\left(t\right)\right]\right)dw_{k}\left(t\right)+Ddv\left(t\right)\\ z\left(t\right)=C_{z}\left(t\right)\bar{x}\left(t\right)+{\bar{C}}_{z}\left(t\right)E\left[\bar{x}\left(t\right)\right] \end{array}\right.$$

where

$$\left\{\begin{array}{c} A_{0}\left(t\right)=\frac{dG(t)}{dt}G^{-1}\left(t\right)+G\left(t\right)A_{0}G^{-1}\left(t\right)\\ {\bar{A}}_{0}\left(t\right)=G\left(t\right){\bar{A}}_{0}G^{-1}\left(t\right)\\ A_{1}\left(t\right)=G\left(t\right)A_{1}G^{-1}\left(t\right)\\ {\bar{A}}_{1}\left(t\right)=G\left(t\right){\bar{A}}_{1}G^{-1}\left(t\right)\\ B(t)=G(t)B\\ C_{0}\left(t\right)=C_{0}G^{-1}\left(t\right)\\ {\bar{C}}_{0}\left(t\right)={\bar{C}}_{0}G^{-1}\left(t\right)\\ C_{1}\left(t\right)=C_{1}G^{-1}\left(t\right)\\ {\bar{C}}_{1}\left(t\right)={\bar{C}}_{1}G^{-1}\left(t\right)\\ C_{z}\left(t\right)=C_{z}G^{-1}\left(t\right)\\ {\bar{C}}_{z}\left(t\right)={\bar{C}}_{z}G^{-1}\left(t\right) \end{array}\right.$$

and $\theta =\frac{2\pi }{w}$. For the numerical application, we take the following:

$$A_{0}=\left[\begin{array}{cccc} -0.841 & 2 & -0.019 & -1\\ 0 & -1.752 & -0.041 & 1\\ 0 & 0 & -2.1 & 1.9\\ 0 & 0 & 0 & -1.2 \end{array}\right],\;{\bar{A}}_{0}=\left[\begin{array}{cccc} 0.06 & -0.028 & 0.028 & 0.02\\ 0.088 & 0.023 & 0.102 & -0.102\\ -0.167 & 0.127 & -0.026 & -0.193\\ 0.051 & 0.026 & 0.011 & 0.005 \end{array}\right],$$

$$A_{1}=\left[\begin{array}{cccc} 0.05 & -0.1 & 0 & -0.05\\ 0.1 & 0.5 & -0.2 & 0.5\\ 0 & 0.25 & 0.5 & 0.05\\ -0.25 & 0 & 0 & -0.5 \end{array}\right],\;{\bar{A}}_{1}=\left[\begin{array}{cccc} -0.095 & 0.182 & -0.036 & -0.075\\ 0.157 & -0.042 & 0.022 & 0.067\\ 0.218 & 0.074 & 0.18 & -0.021\\ -0.077 & 0.126 & 0.154 & 0.128 \end{array}\right],$$

$$B=\left[\begin{array}{cc} -0.06 & 0.03\\ 0.09 & 0.12\\ -0.06 & -0.09\\ 0.06 & 0.03 \end{array}\right],\;C_{0}=\left[\begin{array}{cccc} 1 & 0 & 1 & 0\\ 0 & -1 & 0 & 0 \end{array}\right],$$

$${\bar{C}}_{0}=\left[\begin{array}{cccc} -0.087 & 0.015 & 0.151 & -0.114\\ -0.009 & -0.034 & -0.143 & -0.02 \end{array}\right],$$

$$C_{1}=\left[\begin{array}{cccc} 0.2 & 0.3 & 0 & -0.1\\ 0 & 0 & 0.5 & -0.2 \end{array}\right],\;{\bar{C}}_{1}=\left[\begin{array}{cccc} -0.729 & 0.661 & 0.212 & -0.495\\ -0.156 & 0.495 & -0.113 & -0.104 \end{array}\right],$$

$$D=\left[\begin{array}{cc} 0.1 & 0\\ 0 & 0.1 \end{array}\right],\;C_{z}=\left[\begin{array}{cccc} 1 & 0 & 0 & 1\\ -1 & -1 & 0 & 1 \end{array}\right],\;{\bar{C}}_{z}=\left[\begin{array}{cccc} -1.096 & 0.545 & -0.697 & -0.101\\ -1.317 & 0.95 & -0.182 & -1.794 \end{array}\right]$$

and w=2.

### 6.1. Solution to the Lyapunov-Type Linear Differential Equation (50)

First, we solve the following Lyapunov-like algebraic equation:

(87) $$A_{0}P+PA_{0}^{T}+A_{1}PA_{1}^{T}+BVB^{T}=0$$

This is done by rewriting (87) as follows:

(88) $$\left[\left(I⊗A_{0}\right)+\left(A_{0}⊗I\right)+q_{11}\left(A_{1}⊗A_{1}\right)\right]\mathrm{vec}\left(P\right)=-\mathrm{vec}\left(BVB^{T}\right)$$

where ⊗ is the Kronecker product, and for a (n×p) matrix, $M=\left[\begin{array}{ccc} M^{1} & \cdots & M^{p} \end{array}\right]$, one has $\mathrm{vec}\left(M\right)=\left[\begin{array}{c} M^{1}\\ \vdots \\ M^{p} \end{array}\right]$. Hence, we get $\mathrm{vec}\left(P\right)=-{\left[\left(I⊗A_{0}\right)+\left(A_{0}⊗I\right)+q_{11}\left(A_{1}⊗A_{1}\right)\right]}^{-1}$ $\mathrm{vec}(BVB^{T})$.

The exact unique θ-periodic solution to the Equation (50) is then given via $\Pi_{c}\left(t\right)=G\left(t\right)PG^{T}\left(t\right)$.

### 6.2. Solution to the F-RDE (72)

Here, we follow a similar procedure as in the previous subsection. First, we compute the unique stabilizing solution to the following algebraic Riccati equation (ARE):

(89) $$A_{0}Y+YA_{0}^{T}-\left[YC_{0}^{T}+\tilde{L}\right]{\tilde{R}}^{-1}\left[C_{0}Y+{\tilde{L}}^{T}\right]+\tilde{M}=0$$

where

$$\left\{\begin{array}{c} \tilde{L}=BVD^{T}+A_{1}PC_{1}^{T}\\ \tilde{M}=BVB^{T}+A_{1}PA_{1}^{T}\\ \tilde{R}=DVD^{T}+C_{1}PC_{1}^{T} \end{array}\right.$$

with *P* being the unique solution to (87), using existing stable solvers such as ICARE in MATLAB. The θ-periodic stabilizing solution to the F-RDE (72) is then given via $Y_{s}^{1}\left(t\right)=G\left(t\right)YG^{T}\left(t\right)$, with *Y* being the stabilizing solution to the ARE (89).

**Figure 1:**
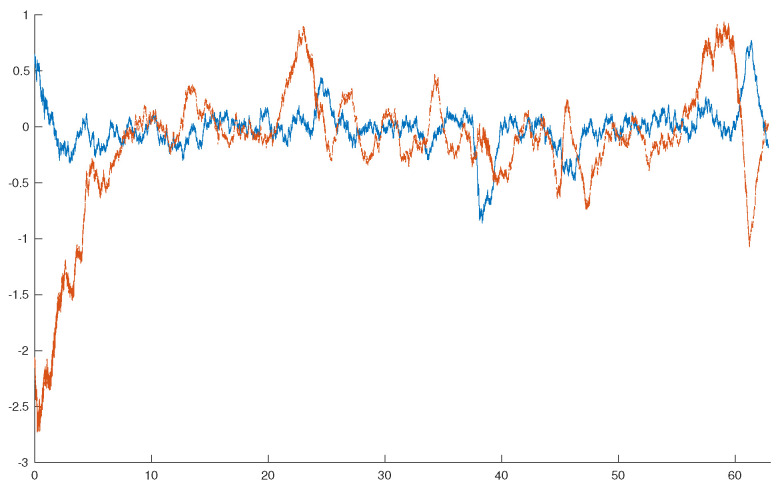
Estimation error: $e\left(t\right)=z\left(t\right)-z_{F}\left(t\right)$.

Figure 1 shows the evolution of the estimation error $e\left(t\right)=z\left(t\right)-z_{F}\left(t\right)$ for one realization of the stochastic processes.

## 7. Conclusions

In this paper, we have considered the problem of optimal $H_{2}$ state estimation for a class of continuous-time, time-varying McKean–Vlasov SDEs. The solution to the considered optimization problem has been expressed in terms of the stabilizing solution to a suitably defined, generalized Riccati differential equation. Some numerical experiments have been provided to show the effectiveness of the proposed method.

## Data Availability

Data is contained within the article.
